# Liver disease, the Swiss army knife of comorbidity: A patient perspective from the grass roots

**DOI:** 10.1002/edm2.110

**Published:** 2020-10-21

**Authors:** Wayne Eskridge

**Affiliations:** ^1^ Fatty Liver Foundation Boise ID USA

## 1 A PATIENT'S VIEW OF THE NASH JOURNEY

When asked to write an article about the patient perspective, it was easy to say yes. Now, looking at this page it is a daunting task. As a cirrhosis patient and Chief Executive Officer (CEO) of the Fatty Liver Foundation, a patient advocacy group, I have my own personal story. My journey from thinking, I was perfectly healthy to facing a terminal illness is common enough but it does not represent what one might describe as the “patient journey.” Through the foundation, we interact with thousands of patients so I hope to provide here a broader glimpse into life as lived by patients.

When we consider patients as a general case in the USA, we see a population that is unwittingly choosing to be ill more often as they age and to die younger and with more difficulty than they expect. Nonalcoholic steatohepatitis (NASH), a disease that was undefined until 1980, has become a leading killer of middle age and older adults. A leading precursor, nonalcoholic fatty liver disease (NAFLD), is steadily increasing in all age groups.

There are many paths to cirrhosis but for the NAFLD patient, obesity is the normal course. The condition of obesity is epidemic with a majority of people in developed countries overweight and nearly 40% clinically obese. The largely asymptomatic course of the disease makes it a silent killer for a large segment of society.

The patient view cannot be condensed to a small set of ideas because the view is ever changing depending on where along the path one stands. From virtually total ignorance in the population at large, to the shock of diagnosis, to knowing far too much about the battle just to live another day, through the shining lottery for life that is transplant, the patient view is endlessly complex. The consequences spread from the patient to the family to society at large as we grapple with the generational tragedy that is this disease of prosperity now epidemic in our society.

## 2 DIAGNOSIS, THE WINDING PATH FROM HEALTHY TO ILL

Through our patient forums, I hear this constantly, “Why didn't my doctor tell me?” A typical variation is “He said I had a fatty liver but it wasn't a big deal.” Then, the classic, “I've never had an abnormal test or any symptoms at all and they say I have cirrhosis.” How is it that so many people face cirrhosis as the first warning of liver disease?

People do not calculate risks well, and they act in ways that are not really logical. Our behaviour is perverse when it comes to things like diet. Society has developed in a way that promotes disease through our industrial food system, constant availability of food and a sedentary lifestyle. We are the creators of our dilemma, but is there a responsibility for physicians to really engage that behavioural problem?

I suppose the profession easily falls victim to the same kind of habit. It is the path of least resistance to give advice in a nonthreatening way. I remember a few decades ago as my blood pressure climbed into the concerning range, and I asked my doctor what that meant. His response was that it seemed just the way all of his patients were these days but I should probably lose some weight.

I think of that at times. My doctor gave me good advice but in a manner that did not engage my concern for my life. The “everyone is doing it” subtext gave me permission to avoid action even though I was routinely engaged with behaviour which damaged my liver. In my case, it was more likely bread than alcohol but I had no idea it was happening.

## 3 THE PHYSICIAN'S ROLE IN ADVANCING LIVER DISEASE

The result of the current standard of care guidelines is to not discover liver disease until symptoms arise. As a consequence, many patients first learn of their problem by being told that they have cirrhosis.

Early‐stage fatty liver disease is often dismissed or minimized by primary care physicians, and patients need only the tiniest excuse to rationalize the warnings. Both parties are at fault in that instance, but a fair question is whether the trusted keeper of our health might have a greater responsibility to be more proactive, before medical complications arise that require intervention. Might a more aggressive wellness message save lives?

Personally, I wonder if I would have engaged more effectively in my health if my doctor had been more emphatic. I cannot answer that as I am also a flawed responder but did I deserve a more honest appraisal of where my lifestyle choices were taking me? An ethics question perhaps, but somewhere in that thicket lies moral hazard, and as a society, we address the issue poorly. Ultimately, it is the patients who pay the price.

There is a school of thought which supports not finding conditions if nothing can be done about it. There is an element of that with NASH. No pill, no point, is effectively the protocol. The fact that weight loss and nutrition are important therapies often does not tip the scale as the training and time requirement for effective support of a nutrition strategy do not fit within our current medical system. Patients want that support and believe that their doctors have that knowledge even though most have neither the knowledge nor the time. A predictable result is that many patients are disappointed, perhaps unfairly, by their healthcare team.

Diagnosis is frequently a difficult process. Primary care processes and procedures do not routinely engage NAFLD at an early stage. Even as the vague early symptoms appear, they do not lead smoothly to considering liver disease. Fatigue has a thousand mothers; vague right upper quadrant discomfort is not a very compelling complaint; gastro intestinal problems could be a lot of things; not an alcoholic OK; blood levels are normal; a little nausea that happens; well bowel habits can change as we age; perhaps a psychologist would be helpful.

It is routine for patients to be referred to multiple specialties as the result of an array of complaints and the process frequently lasts for several years before the focus turns to the liver. I have been surprised at how often patients who are hospitalized and become engaged with their health will examine their medical records and report, “My medical record said I had fatty liver stage 2 five years ago. They never told me that. Why didn't they warn me?” Of course, we know that perhaps the doctor did say something at the time but since fat is benign, until it is not, physicians may make little note of it. Patients become angry at their caregivers in hindsight. We can dismiss it as an easy form of denial but it happens often and as patients we do feel it is wrong.

It is surprisingly common for patients with early‐stage complaints but normal blood levels to be referred to a psychologist. The repeated search for care by patients, absent acute symptoms and no overt alarms, too easily leads to a mental health intervention. When, in the fullness of time, the diagnosis is cirrhosis, a predictable patient response is anger at having been shunted aside as a case of mental illness.

## 4 I HAVE CIRRHOSIS WHAT DO I DO NOW?

This entire journey takes place in the company of Dr Google in our modern world and he is both profoundly evil and a miracle as the patient seeks to understand and to conceive of a future for themselves.

As a society, we do not have a working vocabulary for liver disease. Unlike the more fashionable problems, such as heart disease or cancer where we have mental frameworks to connect with health information, liver disease is largely unknown. Few people know NAFLD or NASH, even in concept. For many people, cirrhosis is a problem for old drunk men and not them. As a result when the words are “you have cirrhosis,” the first response is denial. The “you must be wrong, I'm not an alcoholic” is typical. Without a framework, a description of things like steatohepatitis are lost and the typical patient is disturbed and confused. Without context, even a careful explanation, which is not always provided, is not well understood by the patient.

Patients today have access to their medical information which was invisible to them in the past. Upon diagnosis of a terminal illness, like stage 4 NASH, it quickly becomes apparent that this has likely been coming for a long time. The patient is faced with a vast array of advice in the broader media, and even within the physician community, there is little consistency. Newly diagnosed patients will commonly attempt to lose weight, and it has been shown that most of the dietary strategies will yield some weight loss. Like the broader society, however, most patients do fail and typically regain the weight. For patients who perceive the risk appropriately, this is a source of great concern and many try multiple diets without lasting success.

## 5 THE WEIGHT LOSS DILEMMA

Patients diagnosed with NAFLD or NASH are advised to lose weight. The research is clear that even a 7% weight loss will produce positive histologic changes but that has proven to be a very difficult challenge for many patients.

Patients who struggle with self‐image issues are often further challenged by a sense of failure in the struggle to managed their weight. There is an undercurrent of dissatisfaction with the medical community, and many patients feel that their physician's support is inadequate. It is common for patients to report that they received little useful weight loss advice from their doctor.

Even though diet is foundational to liver health, nutrition is largely a failing enterprise. The patient struggles with following a healthy course in our overfed but poorly served society. The fact that our system discourages the kind of engagement that is required to affect long‐term change can promote a sense of frustration with the healthcare system and defeats a weak resolve to change a lifestyle.

Physicians routinely say lose weight and exercise but for most that is the extent of the advice. There are centres of excellence where there is effective engagement over time which has proven to be the best way for people to learn how to adopt a new lifestyle but those services are the exception and are not available to most people.

This lack of service has produced two alternatives. The diet industry is quite large. It typically sells packaged food products and a community of some kind that helps sustain the subscription model. This strategy is, perhaps unfairly, judged to be a $60 billion dollar failure as most people regain the weight they lose. The alternative, patient support forums, are patient to patient ad hoc communities which provide a great deal of emotional support and how to information. Much of that information is good but there is also a lot of ill‐informed opinion which can be harmful. We have a shortage of generally available, good quality, support for people who seek to learn how to manage their lifestyle in support of better health.

The societal tragedy that is our modern food industry and our ill‐advised daily habits are beyond the scope of this discussion, but for patients, knowledge is power. Even in our food deserts, a knowledgeable patient has an opportunity to live a healthier life if they are well informed. We do not invest in providing the infrastructure and training to foster that and we reap the harvest of disease that we now face. It is ironic that in the age of information, useful nutrition knowledge is not a common currency.

## 6 THE PSYCHIC COST OF CIRRHOSIS

This is the point where patients have serious emotional conflict. Grief at the loss of a loved one is part of the human experience. The coping process is well described starting with shock and denial and ending with acceptance. The important point to understand about a patient who is suddenly told they have a terminal disease, like cirrhosis, and they hear the words “I'm sorry we have no treatment” is that they suffer a death at that moment. It is a death of self. It is the loss of who they believe themselves to be.

The diagnosis means the death of whatever future that person imagined for themselves. Everyone knows they will die but it is mostly philosophical, and they have some sense about how they expect their lives to go. When confronted with the reality of a terminal condition, that imagined future dies and some new journey must take its place.

The result is a mourning process beginning with shock but which quickly leans on denial. “I'm not an alcoholic” is typical. Anger likely follows soon in the “How could someone not tell me?” There is not much bargaining to do but the searching of “There must be something we can do” probably qualifies. A very serious descent into depression is common for those who suffer with end‐stage liver disease. To be fair, it is common with most chronic conditions but can be particularly intense for people who thought they were reasonably healthy but now face the daunting panoply of problems that are characteristic of liver failure.

Most people will come to an acceptance of the situation which may include strategies to improve their lifestyle and consider clinical trials or transplants, but they may just make peace with the inevitable as well. As the incidence of liver disease increases, we also see increasing suicide statistics where liver disease is identified as a triggering event.

## 7 THE SPECIAL ROLE OF THE PHYSICIAN

Once patients understand the long‐term development of this chronic illness, it easily becomes the focus of hindsight. While it may not be entirely fair for patients to feel betrayed, it is important that the profession understands that with information technology readily available today, their special role in patient's lives is damaged by this experience.

It may be that the special role traditionally held by doctors cannot survive our industrialized, process driven healthcare system. As patients, in those moments when we really encounter our fates, we desperately want that relationship. The human values inherent in the healthcare bargain have commonly been a significant factor which caused people to choose healthcare as a profession as well. If those values are to survive, it is important for the profession to recognize that as patients we want our doctors to genuinely care for us. Preserving that depends in part on giving us fair warning even when we would rather not know.

It is disturbing that examples of dysfunction in the management of undiagnosed disease are rife in the patient community. It would belabour the point to make a long list but symptomatic is the example of the general practitioner (GP) whose response to ascites was “You should see someone about that,” when no local gastrointestinal clinics were accepting nonscope referrals and the nearest specialist is 200 miles away.

Easy advice for a patient who has already lost everything financially and depends on Medicaid and Social Security. The result of that encounter in the real world was eventually the emergency room (ER), a week in the intensive care unit (ICU) and finally an emergency transport to a distant transplant centre.

## 8 TRYING TO LIVE WITH END‐STAGE DISEASE

Once symptoms manifest and a diagnosis is in hand, the patient journey has just begun. The road through liver failure hell is extremely difficult. Unlike a heart attack or stroke which has a defining event and a pathway of consequences, liver failure creates a broad spectrum of unwellness that evolves over time. As liver damage increases, organ systems throughout the body suffer and the consequences are many and varied. The course is often a very painful decline with episodes of crisis that goes on for years. Because we have so few liver specialists and much of the physician corp is incompletely trained to manage liver disease, many patients find the journey to be profoundly difficult.

As the disease progresses, patients frequently find themselves in the ER. It is common in the USA for a cirrhosis patient to visit an ER multiple times a year being treated by staff who have limited knowledge of liver disease. Over the years of typical decline, ER visits and hospitalizations become common. A patient suffering advanced fibrosis can easily require the ICU from time to time with the vast burden that places on society and the family.

The general decline and the exacerbation of comorbid conditions produces a panoply of suffering for the patient. Chronic pain, nausea, itching, fatigue and depression are among a long list of problems. To be fair, physicians see pain and suffering every day and there are a lot of ailments that are difficult to manage. There are many ways to die in pain, but most do not have the wide array of dysfunction that results from liver failure. It is the constancy of struggle that has made suicide as a result of liver disease a significant line item in suicide statistics.

## 9 TRANSPLANTS, A SOMETIMES TROUBLING MIRACLE

Modern medicine is a miracle. Transplants are amazing examples of the skill and stewardship that sets the medical profession on a pedestal. There is much to admire in those surgery suites and as patients we grasp at that lifeline. As special as it is, it is also a cruel aspect of cirrhosis. It is the oasis in a thirsty desert that only a few can reach but all can see. A modest fraction of those in need can even apply, and most will not catch the winning ticket.

The transplant journey is a strange stew for patients. The rules have a certain perverse logic about how the privilege of life is earned but there is a cost. Spoken or not, patients know that the trick is be sicker than your competitor. Imagine the conflict for the wife who says, “I'm so upset, my husband's MELD (model for end stage liver disease) score came down but he is so sick. I am so afraid he won't get a chance if he doesn't get sicker, but he is so close to getting too sick.”

We do thousands of transplants each year but tens of thousands of patients are diagnosed with cirrhosis. Might those vast sums being spent to provide miracles for a few, be more productively spent on the larger population? Might we be better served investing in prevention or early engagement? Difficult questions for patients and for the industry. How one views the moral case depends entirely on where you sit on competing scales of need and opportunity.

## 10 THE IMMENSE SOCIAL BURDEN OF LIVER DISEASE

The baseline of chronic illness with frequent critical episodes brings with it perhaps the greatest tragedy of this disease. As a society, we are incompetent at dealing with chronic serious long‐term decline. As a patient becomes unwell, they require increasing care which commonly falls on a spouse or family member. The result is that two productive earners are taken out of the workforce, and many family units are ground inexorably into poverty.

Societal resources are poorly supplied, badly distributed, and the quality of what support exists varies widely. Most families are ill‐equipped to deal with the expense and continuous care that goes with the slow dance with the angel of death that is end‐stage liver disease. Medicine engages at the points of crisis but in between is the day by day struggle that burdens the soul of patients and caregivers as they seek to get help for chronic problems from a system geared to crisis.

From the perspective of the foundation, the blind eye turned to the plight of those too well off to be helped by the safety net and too poor to cope with the cost of modern medicine, even with insurance, is a real injustice. Services are more available near major medical centres but imagine being the patient in a more distant community whose general practitioner (GP) tells them not to come back but there are no local physicians who manage liver disease. Or maybe being sick and the few offices who might consider taking you are scheduled out months. Return with me now to the emergency room where you cannot be helped until you crash. You can come in, of course, but ER docs are poorly equipped to deal with you. Smaller hospitals do not deal well with the need for regular paracentesis or consider not being given needed albumin after a procedure and ending up in the ICU with a bleed and all of this increasing the financial burdens you face.

As the burden of disease moves into every younger cohorts, liver disease is now the number 4 killer of the 45‐ to 54‐year‐olds, the societal costs rise quickly. Now, instead of ageing parents being cared for by mature children, we have parents dying of cirrhosis with young children still at home. If that is not a sufficient harbinger of the crisis before us to prompt a meaningful response, thoughtful management may have left the building.

## 11 THE PROMISE OF DRUG THERAPY

As current patients, we pray for therapy of some kind. For the advanced fibrosis patient, there is a powerful yearning for relief of some kind and the news of research is tantalizing. The reports have a dark side, however, as we know how slowly those wheels turn. There is a burden attached to hearing of all the research being done but also believing that any help will come too late for you.

From the foundation's perspective, while a successful therapy would be a godsend, the search also detracts from the important fact that for many patients, it would have been possible to never have needed the drug at all. Effective drug treatment is critical, but it should be the second therapy not the first.

## 12 ON THE OTHER HAND, THERE ARE SUCCESSFUL PATIENTS

It is inappropriate to just highlight the dark side of the patient experience. There are shining examples of a better way. The disturbing dangers of social media have also spawned large peer‐to‐peer self‐help groups of patients who support each other in this journey.

In spite of the growing threat, we see more success stories every day. The body of testimony of people who are achieving success and reducing the severity of their liver disease through diet and exercise is growing. We do know that in many cases, concerted thoughtful action by the patient can halt the progression of the disease and even result in remodelling of the fibrosis over time. It is a case by case situation, but early intervention unquestionably has the ability to greatly improve the prognosis for patients. Whether we, as a society, can engage the disease earlier and provide the kinds of support that are required remains to be seen.

Managing a slow, progressive disease like NASH is a difficult challenge in the context of our modern, highly, industrialized society. The often counterproductive actions by government, and misleading marketing are hazards, but the blossoming of patient advocacy and the wellness movement are hopeful signs. As patients become more aware of their risks and better understand how to deal with obesity and all of its comorbidity there is hope for a better outcome long term. It will not be a simple journey but progress is possible.

The development of engaged patients working together in self‐help peer‐to‐peer groups through the internet is among the most encouraging developments today. Community and a shared journey are vital aspects of our lives and much of that is stripped away by chronic disease. Many patients retreat and become invisible to society as they struggle day to day. The stigma, the pain, and the fatigue of the constant battle leave no room for engagement in the manner of those not yet ill. The connections that are possible through these ad hoc groups become a lifeline and are a genuine bright spot made possible by patients themselves.

## 13 WHAT DO PATIENTS NEED

The most important need today is for effective noninvasive wellness screening and early referral by the patient care community to liver disease centric support. Early detection allows management of the condition without engagement of the hepatology specialty.

That support system is best imagined as a multispecialty care team focused on nutrition, mental health and social engagement. The management of obesity and diet is a fundamental societal element so effective engagement must include, at a minimum, the primary caregiver but necessarily impacts the family and society broadly. The epidemic of liver disease that we face is rooted in the development of food strategies and cultural practices that tend towards obesity and illness long term. Solving the problem is not just the province of the physician, of course, but the profession also cannot look away and wait for a drug when diet and behaviour are better treatment.

Medicine should champion the creation of care teams for the not yet ill. Advancing liver disease should be intercepted as early as possible and lifestyle support and nutrition education offered intensively and as early as possible to family units.

## 14 HOW DO WE JUSTIFY TREATING WITHOUT SYMPTOMS

The narrow argument made by the medical establishment is that since we cannot predict who will develop cirrhosis we cannot intervene early. The foundation believes that analysis is too narrow and lacks the proper balance.

We know that liver disease is comorbid with a host of other medical conditions. If we consider the benefit to society that would result from changing long‐term destructive behaviour, it far exceeds the cost of intervention. We believe that the looming threat of liver disease carries with it the opportunity for the creation of teachable moments for a broad swath of society that is steadily developing a host of other disease. If, by focusing on liver health, we create spinoff benefit across the spectrum of lifestyle driven disease, we will have performed a great service to ourselves and our posterity.

## CONFLICT OF INTEREST

Nothing to declare.

